# Fed-batch production of MCL-PHA with elevated 3-hydroxynonanoate content

**DOI:** 10.1186/2191-0855-3-50

**Published:** 2013-08-29

**Authors:** Xuan Jade Jiang, Zhiyong Sun, Juliana A Ramsay, Bruce A Ramsay

**Affiliations:** 1 Chemical Engineering, Queen's University, Kingston, ON K7L 3N6, Canada; 2 Polyferm Canada Inc., RR1, Harrowsmith, ON K0H 1V0, Canada

**Keywords:** PHA, Fed-batch, Acrylic acid, *Pseudomonas putida*, β-oxidation inhibition

## Abstract

With no inhibition of β-oxidation, *Pseudomonas putida* KT2440 produces medium-chain-length poly(3-hydroxyalkanoate) (MCL-PHA) with approximately 65 mol% 3-hydroxynonanoate (HN) from nonanoic acid. Production of PHA with higher HN content and an adjustable monomeric composition was obtained using acrylic acid, a fatty acid β-oxidation inhibitor, together with nonanoic acid and glucose as co-substrates in fed-batch fermentations. Different monomeric compositions were obtained by varying the feeding conditions to impose different specific growth rates and inhibitor feed concentrations. At a nonanoic acid: glucose: acrylic acid feed mass ratio of 1.25: 1: 0.05 and a specific growth rate of 0.15 h^-1^, 71.4 g L^-1^ biomass was produced containing 75.5% PHA with 89 mol% HN at a cumulative PHA productivity of 1.8 g L^-1^ h^-1^.

## Introduction

Poly(3-hydroxyalkanoates) (PHAs) are a family of biodegradable, and non-cytotoxic biopolyesters produced from renewable resources. Certain types of PHAs, such as poly(3-hydroxybutyrate-co-3-hydroxyvalerate) (P(HB-HV)) and poly(3-hydroxybutyrate-co-3-hydroxyhexanoate) (P(HB-HHx)), have been recognized as substitutes for petroleum-based thermoplastics in various applications and have been or are planned to be produced commercially (Philip et al. 2007; Poirier et al. 1995).

In contrast to short-chain-length PHAs (SCL-PHAs) such as P(HB-HV) and SCL-MCL-PHAs such as P(HB-HHx), medium-chain-length PHAs (MCL-PHAs) are thermoplastic elastomers with a much higher elongation-to-break (Van der Walle et al. 2001). They also have lower melting temperatures, are less crystalline and crystallize more slowly (Gagnon et al. 1992; Gross et al. 1989; Marchessault et al. 1990). Most bioreactor scale production of MCL-PHAs have used structurally related MCL carbon substrates, such as octane (Hazenberg and Witholt 1997), nonanoic acid (Sun et al. 2007), and oleic acid (Lee et al. 2000). Although Liu et al. (2011) and Chung et al. (2011) effectively showed MCL homopolymer production by extensive deletion of genes in the β-oxidation pathway, this approach does not allow tailored control of the monomeric composition. We have recently demonstrated that using a combination of a PHA structurally related substrate (e.g. nonanoic or octanoic acid), a structurally unrelated substrate (e.g. glucose), and a fatty acid β-oxidation inhibitor (e.g. acrylic acid), a series of poly(3-hydroxynonanoate-co-3-hydroxyheptanoate) (PHN) with adjustable 3-hydroxynonanoate (HN) content (69 to 96 mol%) or poly(3-hydroxyoctanoate-co-3-hydroxyhexanoate) (PHO) with adjustable 3-hydroxyoctanoate (HO) content (88 to 98 mol%) can be produced (Jiang et al. 2012). It was also demonstrated that the PHA thermal and mechanical properties improved as the amount of the dominant monomer increased. Substrate utilization efficiency also improved with a yield of fatty acid to PHA conversion as high as 0.91 g g^-1^. These results were produced in chemostat but this cultivation technique is not used commercially. In order to be of commercial interest, this novel approach to MCL-PHA production must be shown to be applicable to fed-batch culture.

The objective of this study was to develop a methodology for controlling the monomeric composition of MCL-PHA in efficient fed-batch fermentations. Specifically, the production of PHN copolymers with different HN content was investigated by controlling the specific growth rate and the β-oxidation inhibitor concentration in the feed. The study also employed glucose and nonanoic acid co-feeding to meet the requirements of both cell growth and PHA accumulation, respectively.

## Materials and methods

### Microorganism and growth medium

*Pseudomonas putida* KT2440 (ATCC 47054) was maintained on nutrient agar plates at 4°C. The inoculum medium for all fermentations contained per liter: (NH4)_2_SO_4_ 4.70 g, MgSO_4_ · 7 H_2_O 0.80 g, Na_2_HPO_4_ · 7 H_2_O 12.00 g, KH_2_PO_4_ 2.70 g, nutrient broth 1.00 g, glucose 9.00 g. The initial culture medium contained per liter: (NH_4_)_2_SO_4_ 4.70 g, MgSO_4_ · 7H_2_O 0.80 g, Na_2_HPO_4_ · 7H_2_O 18.0 g, KH_2_PO_4_ 4.05 g, trace element solution 10 mL. The trace element solution contained per liter: FeSO_4_ · 7H_2_O 10.0 g, CaCl_2_ · 2H_2_O 3.0 g, ZnSO_4_ · 7H_2_O 2.2 g, MnSO_4_ · 4H_2_O 0.5 g, H_3_BO_3_ 0.3 g, CoCl_2_ · 6H_2_O 0.2 g, Na_2_MoO_4_ · 2H_2_O 0.15 g, NiCl_2_ · 6H_2_O 0.02 g and CuSO_4_ · 5H_2_O 1.00 g. Nonanoic acid (98%, Spectrum Chemicals) was fed separately in its pure form as it is immiscible in aqueous media. Acrylic acid (Sigma-Aldrich) was added to a glucose (99.5%, Sigma-Aldrich) solution of 240 g L^-1^. Feeding ratios of nonanoic acid (NA), glucose (G) and acrylic acid (AA) at 1.25: 1: 0.01 and 1.25: 1: 0.05 (w/w) were tested. Nitrogen was provided as 14% (w/v) ammonia solution and also served as the base for pH control. In case of nutrient depletion, supplemental solutions of trace elements with the above composition and a phosphate solution containing 36 g L^-1^ Na_2_HPO_4_ · 7H_2_O and 8.1 g L^-1^ KH_2_PO_4_ were prepared. Antifoam 204 (Sigma-Aldrich) was added to nonanoic acid (1% v/v) and manually injected through a sterile septum when required.

### Fermentation conditions

The inoculum was grown in three 500 mL shake flasks (100 mL medium in each flask) at 28.0 ± 1°C and 200 rpm overnight. The first two fermentations were conducted in a 7 L MBR stirred tank bioreactor (Bioreactor-AG, Switzerland) with a 5 L working volume. The third fermentation was done in a 5 L Minifors bioreactor (Infors-HT, Bottmingen, Switzerland) with a 3 L working volume. The cultivation temperature was 28.5 ± 1°C and the pH was controlled at 6.85 ± 0.05 using 14% (w/v) ammonia solution. Dissolved oxygen was measured with an Ingold polarographic probe and maintained above 30% air saturation by adjusting the agitation speed and the mixture of air and oxygen flow via mass flow controllers to a total gas flow at 1 vvm. The dissolved oxygen data were acquired by a LabVIEW 6.1 (National Instrument) program. Nonanoic acid and glucose feeding was controlled via separate peristaltic pumps by the LabVIEW program based on the mass of each reservoir.

### Substrate feeding and control methods

The specific growth rate was controlled at 0.25 h^-1^ or 0.15 h^-1^ by exponentially feeding the carbon sources to be the growth-limiting nutrient. It was estimated that 1 g L^-1^ biomass would be produced from 1.6 g L^-1^ total carbon sources in the initial fermentation medium. The cumulative mass of carbon substrates S_t_ (g) to be fed at time t (h) was calculated based on exponential cell growth (X_t_, g) expressed in the Equation below.

(1) $$S_{t}=\frac{X_{t}}{Y_{X/C}}=\frac{X_{0}}{Y_{X/C}}\cdot \left(e^{\mu t}-1\right)$$

where X_0_ (g) is the estimated biomass at the beginning of the feeding; *μ* (h^-1^) is the desired specific growth rate; and *Y*_*X/C*_ is the yield (g g^-1^) of biomass from the mixture of carbon substrates which was 0.66 g g^-1^, experimentally determined from continuous fermentation by feeding nonanoic acid, glucose and acrylic acid at a mass ratio of 1.25: 1: 0.05 at a specific growth rate of 0.25 h^-1^ (Jiang et al. 2012).

The mass of each carbon source required at time t was calculated according to the pre-defined ratio of the two substrates as follows:

(2) $$S_{t-\mathrm{NA}}=S_{t}\cdot f_{\mathrm{NA}}$$

(3) $$S_{t-G}=S_{t}\cdot f_{G}$$

The feeding ratio of nonanoic acid to glucose in this study was 1.25: 1 (w/w). Therefore, the mass fraction of nonanoic acid (*f*_NA_) and that of glucose (*f*_G_) in the total carbon source were 0.56 and 0.44, respectively.

Exponential substrate feeding began after a lag phase of approximately 5 h. Fermentations with a specific growth rate of 0.25 h^-1^ were conducted only under exponential feeding. However, in an effort to avoid nonanoic acid and acrylic acid overfeeding, exponential feeding at 0.15 h^-1^ was conducted for 23.3 h before changing to a constant feed rate of 8 g L^-1^ h^-1^.

### Analytical procedures

Biomass concentration was determined gravimetrically from duplicate samples of 10 mL culture broth which were centrifuged at 6,000 × *g* for 15 min, washed and lyophilized. Sample supernatants were analyzed for the concentrations of residual nutrients and acrylic acid. Glucose was measured colorimetrically after reacting with 4-hydroxybenzoic hydrazide under alkaline condition (Lever 1972). Nonanoic acid was methylated in acidified methanol (Ramsay et al. 1991) and analyzed by a CP3900 Varian GC equipped with a flame ionization detector. Phosphate was measured based on the reduction of phosphomolybdate to molybdene blue (Clesceri et al. 1999). Ammonium was determined by the phenol-hypochlorite method (Weatherburn 1967). Acrylic acid was assayed by Hewlett-Packard GC equipped with a Cabowax®-PEG column after acidification with one tenth volume of 2 N hydrochloric acid (Qi et al. 1998).

PHA content and composition in the dry biomass samples were determined by methanolysis in 2 mL chloroform and 1 mL methanol which contained sulfuric acid (15% v/v) as acidifying agent and benzoic acid (0.2% w/v) as internal standard at 100°C for 4 h. After which, 1 mL distilled water was vigorously mixed on a Fisher Vortex and left overnight for phase separation. One *μ*L of the chloroform phase was injected into CP3900 Varian GC at a split ratio of 20. The injector and detector were maintained at 250 and 275°C, respectively. The oven heating profile was: initial 90°C for 0.5 min, 5°C min^-1^ to 95°C and hold for 0.5 min, 30°C min^-1^ to 170°C and hold for 2.5 min. The PHA standard was prepared by acetone extraction and methanol precipitation followed by three cycles of extraction and precipitation, as described by Jiang et al. (2006) and the monomeric composition characterized by GC and proton nuclear magnetic resonance at room temperature in a Bruker Avance 200 spectrometer using deuterated-chloroform containing 20 mg mL^-1^ PHA.

## Results

### Co-feeding nonanoic acid, glucose, and acrylic acid at a mass ratio of 1.25: 1: 0.01 and a *μ* of 0.25 h^-1^

**Figure 1:**
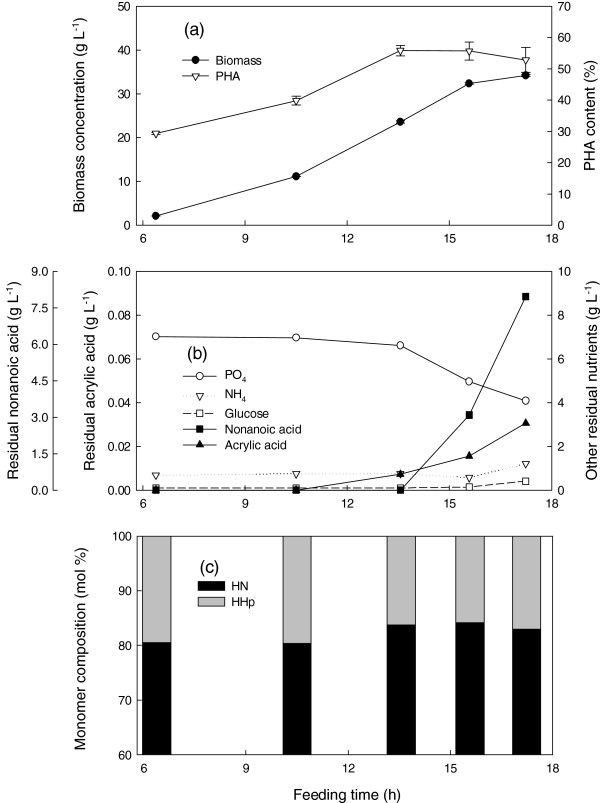
**Cultivation of *P. putida *KT2440 by feeding nonanoic acid, glucose and acrylic acid at a mass ratio of 1.25: 1: 0.01 and a specific growth rate of 0.25 h**^**-1 **^**in fed-batch fermentation at 28.5 ± 1°C, pH 6.85 ± 0.05, DO > 30%.** Fermentation data **(a)** and **(b)**, polymer composition **(c)**. Foam was controlled by manual injection of antifoam 204 in 1% (v/v) nonanoic acid as required. HN,3-hydroxynonanoate; HHp, 3-hydroxyheptanoate. Symbols are the average of two samples and error bars represent the range of the average.

Co-feeding nonanoic acid (NA), glucose (G), and acrylic acid (AA) at a mass ratio of 1.25: 1: 0.01 and a specific growth rate of 0.25 h^-1^ produced 34 g L^-1^ dry biomass containing a maximum of 56% PHA (Figure 1a). Phosphate and ammonium were controlled at levels that had been previously shown to be sufficient but not inhibitory to cell growth (Sun et al. 2007). Before 13.6 h, nonanoic acid and glucose concentrations were very low, indicating that the carbon source was the only limiting factor (Figure 1b). Uncontrollable foaming occurred at 15.8 h, accompanied by accumulation of acrylic and nonanoic acids in the reactor but glucose was entirely consumed. During the early stages of cultivation, the PHA contained 81 mol% HN which increased slightly to 84 mol% (Figure 1c), much more than the 65 mol% HN produced without acrylic acid.

### Co-feeding nonanoic acid, glucose, and acrylic acid at a mass ratio of 1.25: 1: 0.05 and a *μ* of 0.25 h^-1^

**Figure 2:**
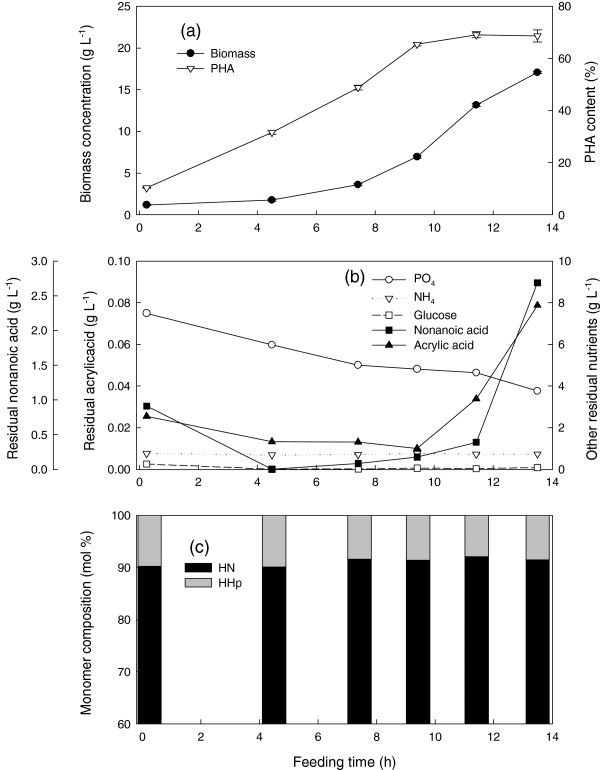
**Cultivation of *P. putida *KT2440 by feeding nonanoic acid, glucose and acrylic acid at a mass ratio of 1.25: 1: 0.05 and a specific growth rate of 0.25 h**^**-1 **^**in fed-batch fermentation at 28.5 ± 1°C, pH 6.85 ± 0.05, DO > 30%.** Fermentation data **(a)** and **(b)**, polymer composition **(c)**. Foam was controlled by manual injection of antifoam 204 in 1% (v/v) nonanoic acid as required. HN, 3-hydroxynonanoate; HHp, 3-hydroxyheptanoate. Symbols are the average of two samples and error bars represent the range of the average.

In an attempt to further increase the HN content, the culture conditions were kept the same as above except that the amount of acrylic acid was increased by a factor of five. The results were similar to what is shown in Figure 1 except that the residual nonanoic acid and acrylic acid accumulated earlier (starting at 12.4 h instead of 15.8 h). Again, there was uncontrollable foaming and only 17 g L^-1^ final biomass was produced (Figure 2a, b). However, the PHA content increased from 56 to 64% with about 90 mol% HN (Figure 2c).

### Co-feeding nonanoic acid, glucose, and acrylic acid at a mass ratio of 1.25: 1: 0.05 and a *μ* of 0.15 h^-1^

**Figure 3:**
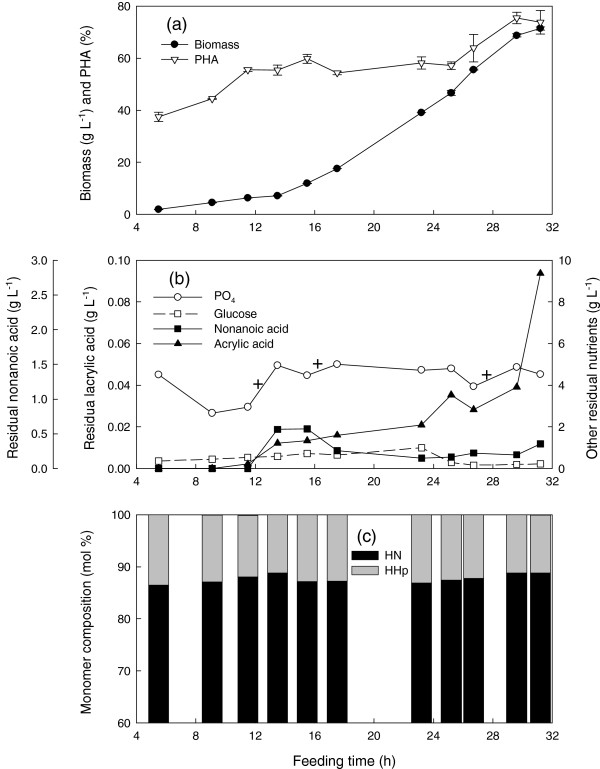
**Cultivation of *P. putida *KT2440 by feeding nonanoic acid, glucose and acrylic acid at a mass ratio of 1.25: 1: 0.05 and a specific growth rate of 0.15 h**^**-1 **^**in fed-batch fermentation at 28.5 ± 1°C, pH 6.85 ± 0.05, DO > 30%.** Fermentation data **(a)** and **(b)**, polymer composition **(c)**. Foam was controlled by manual injection of antifoam 204 in 1% (v/v) nonanoic acid as required. HN, 3-hydroxynonanoate; HHp, 3-hydroxyheptanoate. 20 mL supplemental phosphate solution was added at the times indicated by “+” above the phosphate curve. Symbols are the average of two samples and error bars represent the range of the average.

In order to avoid both acrylic acid and nonanoic acid accumulation, the feeding program was adjusted to achieve a lower *μ* of 0.15 h^-1^ at a NA: G: AA feeding ratio of 1.25: 1: 0.05 for the first 23.3 h followed by a constant feed rate of 8 g L^-1^ h^-1^. Under these conditions, a final biomass concentration of 71.4 g L^-1^ was achieved (Figure 3a). The PHA content increased in two steps, from 0 to 59.8% followed by a 10 h plateau, until the change to a constant feed rate when there was a second increase from 58.1 to75.5%.

There was constant foaming from the beginning of the fermentation. This became more severe at 12 h. At this time, the phosphate (20 mL) and trace element solutions (30 mL) were added. Antifoam was added dropwise and the foam disappeared after about 30 min. Phosphate was maintained at non-limiting levels while ammonium was automatically controlled to be in the range of 1 ~ 1.5 g L^-1^ (Figure 3b) as in the previous two fermentations. The glucose concentration was always slightly above zero. There was a slight increase in the nonanoic acid concentration between 12 h and 16 h, but its concentration dropped after 16 h and remained below 0.5 g L^-1^ until near the end of the fermentation. Despite a supply of 1 vvm pure oxygen, the dissolved oxygen dropped to zero at 29.6 h and remained there for the duration of the fermentation. PHN containing about 88 mol% HN was obtained.

### Comparison of the three fed-batch fermentations

**Figure 4:**
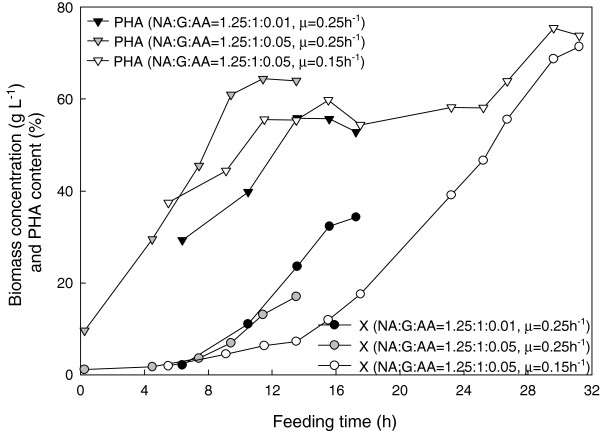
**Effect of feed ratio and feed rate on biomass and PHA production in *P. putida *KT2440 when co-feeding nonanoic acid and glucose at together with acrylic acid.** NA, nonanoic acid; G, glucose; AA, acrylic acid; X, biomass.

**Figure 5:**
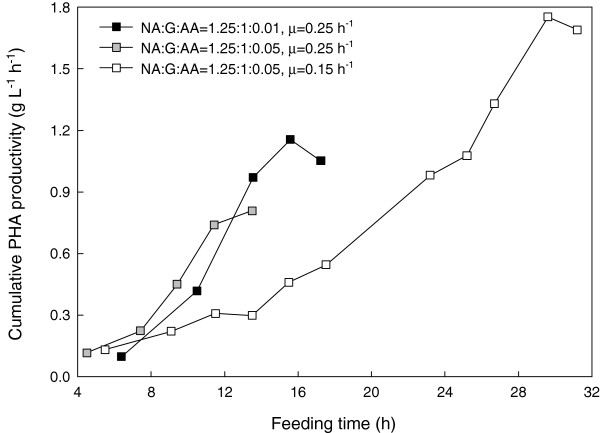
**Cumulative PHA productivity in *P. putida *KT2440 under nonanoic acid and glucose co-feeding at the presence of acrylic acid in fed-batch fermentations.**

Regardless of the acrylic acid concentration, feeding to maintain a specific growth rate of 0.25 h^-1^ produced the same biomass trend (Figure 4) until foaming occurred, ending the fermentations. However, at a lower specific growth rate (0.15 h^-1^), higher biomass and PHA content were eventually achieved. While cumulative PHA productivity (PHA in g L^-1^ divided by total fermentation time) increased more quickly at a higher specific growth rate, the highest cumulative productivity of 1.8 g L^-1^ h^-1^ was obtained with a combination of the higher acrylic acid concentration and lower growth rate (Figure 5).

## Discussion

Control of the monomeric composition of MCL-PHA in a fed-batch fermentation using a β-oxidation inhibitor is novel and challenging. Bacterial cultivation using fatty acid substrates in the presence of acrylic acid has been shown to produce poor growth and MCL-PHA accumulation both in our chemostat studies (Jiang et al. 2012) and in the literature (Huijberts et al. 1994; Qi et al. 1998; Ward and O’Connor 2005). This is because β-oxidation is the only mechanism of energy production from aliphatic fatty acids. Thus, the strategy of co-feeding a carbon and energy source (glucose in this study) and a PHA precursor (nonanoic acid in this study) is essential to obtain a high cell density with high PHA content.

As the acrylic acid concentration increased in the co-substrate feed, at the same feed rate (*μ* = 0.25 h^-1^), the accumulation of MCL-PHA increased as well as the proportion of HN monomers from 65 mol% (no acrylic acid) to greater than 92 mol% (at NA: G: AA = 1.25: 1: 0.05) (Table 1). In an earlier study (Jiang et al. 2012), we showed that increasing HN monomers had no significant effect on the weight average molecular weight but did affect thermal and mechanical properties. The increase in the HN content probably reflects increasing β-oxidation inhibition with the increasing acrylic acid concentration. Fed-batch fermentation was able to obtain the same inhibition as chemostat cultivation as demonstrated by the similar monomeric composition and cumulative PHA productivity (Table 1) at the same growth rate (*μ* =0.25 h^-1^) and NA: G: AA feeding ratio (1.25: 1: 0.05). However, in contrast to a continuous or batch cultivation where nutrients and other additives are either at steady state concentrations or gradually consumed, substrates in fed-batch fermentations may accumulate if they are consumed more slowly than the feed rate. Accumulation of toxic substances could be harmful since it may lead to cell death and uncontrollable foaming. About 3 ~ 4 g L^-1^ of nonanoic acid (Sun et al. 2006) and as little as 0.1 g L^-1^ acrylic acid are toxic to *P. putida* KT2440. In this study, the use of acrylic acid is even more challenging since nonanoic acid accumulation was accelerated by β-oxidation inhibition and occurred very quickly as seen in the two fermentations at the higher feed rate (*μ* = 0.25 h^-1^). At a lower feed rate (*μ* = 0.15 h^-1^ followed by linear feeding), although there were two minor foaming events at 12 and 23 h accompanied by a noticeable but lower level of nonanoic and acrylic acid accumulation, foaming was controllable. This resulted in a longer fermentation with much higher biomass production (71.4 g L^-1^), higher MCL-PHA accumulated (75.5%) with high HN content (about 89 mol%) and the best MCL-PHA productivity (1.8 g L^-1^ h^-1^) and yield of PHA from NA (0.78 g g^-1^) (Table 1). This may be improved further by using a decaying substrate feeding strategy (Maclean et al. 2008).

**Table 1:** **Comparison of fermentations producing PHN using *P. putida *KT2440**^**4**^

**Specific growth rate (h**^**-1**^**)**	**NA:G:AA**^**1**^**feeding ratio (w/w/w)**	**Fermentation type**	**Biomass** **(gL**^**-1**^**)**	**PHA (%)**	**HN (mol%)**	**HHp (mol%)**	***Yx/c***^**2**^**(g g**^**-1**^**)**	***Y***_***PHA/NA***_^**2**^**(g g**^**-1**^**)**	**Cumulative PHA productivity**^**3**^ **(g L**^**-1**^ **h**^**-1**^**)**	**Reference**
0.25	1:1:0	Fed-batch	71.0	56.0	65.0	35.0	0.62	0.66	1.4	Sun et al. 2009
0.25	1.25:1:0.05	Chemostat	5.8	51.7	91.3	8.7	0.66	0.61	0.7	Jiang et al. 2012
0.25	1.25:1:0.01	Fed-batch	34.3	55.7	84.3	15.7	0.62	0.68	1.2	This study
0.25	1.25:1:0.05	Fed-batch	17.1	64.4	92.2	7.8	0.53	0.68	0.8	This study
0.15	1.25:1:0.05	Fed-batch	71.4	75.5	88.9	11.1	0.62	0.78	1.8	This study

^1^*NA* nonanoic acid, *G* glucose, *AA* acrylic acid, *HN* 3-hydroxynonanoate, *HHp* 3-hydroxyheptanoate.

^2^*Y*_*X/C*_ yield of biomass from total carbon substrate, *Y*_*PHA/NA*_ yield of PHA from nonanoic acid.

^3^ Calculated as PHA in g L^-1^ divided by total fermentation time.

^4^ Biomass, PHA content, HN and HHp percentage, and PHA productivity were reported as the highest values during the fermentations, while *Y*_*X/C*_ and *Y*_*PHA/NA*_ were reported as the slopes of the trend line that was drawn from all points of each fermentation.

Whether it is metabolized or not, acrylic acid consumption was linearly related to cell growth, in a manner similar to nonanoic acid consumption (Figures 1, 2 and 3). Since it is continuously taken up by the cells, the feeding of acrylic acid should be proportional to cell growth in order to impose a constant level of inhibition and thus a constant PHA monomeric composition. The combination of an appropriate concentration of the β-oxidation inhibitor and a growth rate which avoids toxic accumulation of both nonanoic and acrylic acid enhanced growth and PHA accumulation as well as controlled the monomeric composition. This is the first report of the use of a β-oxidation inhibitor in high-cell-density fed batch production of MCL-PHA.

## Competing interests

The authors hold a patent partially based on the paper (US pat no. 8273852).

## Authors’ contribution

XJJ carried out fermentations and analyses, XJJ, ZS, JAA and BAA were involved in data presentation, interpretation and writing of manuscript. All authors read and approved the final manuscript.

## References

[B1] Chung AL Jin HL Huang LJ Ye HM Chen JC Wu Q Chen GQ Biosynthesis and characterization of poly(3-hydroxydodecanoate) by ß-oxidation inhibited mutant of *Pseudomonas entomophila* L48 Biomacromolecules 2011 3 3559 3566 10.1021/bm200770m 21838281

[B2] Clesceri LS Greenberg AE Eaton AD Standard methods for the examination of water and wastewater 1999 20 Washington, DC: American Public Health Association

[B3] Gagnon KD Lenz RW Farris RJ Fuller RC Crystallization behavior and its influence on the mechanical properties of a thermoplastic elastomer produced by *Pseudomonas oleovorans* Macromolecules 1992 3 3723 3728 10.1021/ma00040a018

[B4] Gross RA Demello C Lenz RW Brandl H Fuller RC Biosynthesis and characterization of poly(beta-hydroxyalkanoates) produced by *Pseudomonas oleovorans* Macromolecules 1989 3 1106 1115 10.1021/ma00193a018

[B5] Hazenberg W Witholt B Efficient production of medium-chain-length poly(3-hydroxyalkanoates) from octane by *Pseudomonas oleovorans*: economic considerations Appl Microbiol Biotechnol 1997 3 588 596 10.1007/s002530051100

[B6] Huijberts GNM Derijk TC Dewaard P Eggink G C-13 nuclear-magnetic-resonance studies of *Pseudomonas putida* fatty-acid metabolic routes involved in poly(3-hydroxyalkanoate) synthesis J Bacteriol 1994 3 1661 1666 8132461 10.1128/jb.176.6.1661-1666.1994 PMC205252

[B7] Jiang X Ramsay JA Ramsay BA Acetone extraction of mcl-PHA from *Pseudomonas putida* KT2440 J Microbiol Methods 2006 3 212 219 10.1016/j.mimet.2006.03.015 16753235

[B8] Jiang X Sun Z Marchessault RH Ramsay J Ramsay B Biosynthesis and properties of medium-chain-length polyhydroxyalkanoates with enriched content of the dominant monomer Biogeosciences 2012 3 2926 2932 10.1021/bm3009507 22871146

[B9] Lee SY Wong HH Choi JI Lee SH Lee SC Han CS Production of medium-chain-length polyhydroxyalkanoates by high-cell-density cultivation of *Pseudomonas putida* under phosphorus limitation Biotechnol Bioeng 2000 3 466 470 10.1002/(SICI)1097-0290(20000520)68:4<466::AID-BIT12>3.0.CO;2-T 10745215

[B10] Lever M New reaction for colorimetric determination of carbohydrates Anal Biochem 1972 3 273 279 10.1016/0003-2697(72)90301-6 5031119

[B11] Liu Q Luo G Zhou XR Chen GQ Biosynthesis of poly(3-hydroxydecanoate) and 3-hydroxydodecanoate dominating polyhydroxyalkanoates by β-oxidation pathway inhibited *Pseudomonas putida* Metabolic Eng 2011 3 11 17 10.1016/j.ymben.2010.10.004 20971206

[B12] Maclean H Sun Z Ramsay J Ramsay B Decaying exponential feeding of nonanoic acid for the production of medium-chain-length poly(3-hydroxyalkanoates) by *Pseudomonas putida* KT2440 Can J Chem 2008 3 564 569 10.1139/v08-062

[B13] Marchessault RH Monasterios CJ Morin FG Sundararajan PR Chiral poly(beta-hydroxyalkanoates) - an adaptable helix influenced by the alkane side-chain Int J Biol Macromol 1990 3 158 165 10.1016/0141-8130(90)90068-L 2078532

[B14] Philip S Keshavarz T Roy I Polyhydroxyalkanoates: biodegradable polymers with a range of applications J Chem Technol Biotechnol 2007 3 233 247 10.1002/jctb.1667

[B15] Poirier Y Nawrath C Somerville C Production of polyhydroxyalkanoates, a family of biodegradable plastics and elastomers in bacteria and plants Biogeosciences 1995 3 142 150 10.1038/nbt0295-142 9634754

[B16] Qi Q Steinbuchel A Rehm BH Metabolic routing towards polyhydroxyalkanoic acid synthesis in recombinant *Escherichia coli* (fadR): inhibition of fatty acid beta-oxidation by acrylic acid FEMS Microbiol Lett 1998 3 89 94 9785457 10.1111/j.1574-6968.1998.tb13212.x

[B17] Ramsay BA Saracovan I Ramsay JA Marchessault RH Continuous production of long-side-chain poly-beta-hydroxyalkanoates by *Pseudomonas oleovorans* Appl Environ Microbiol 1991 3 625 629 16348427 10.1128/aem.57.3.625-629.1991 PMC182769

[B18] Sun Z Ramsay JA Guay M Ramsay BA Automated feeding strategies for high-cell-density fed-batch cultivation of *Pseudomonas putida* KT2440 Appl Microbiol Biotechnol 2006 3 423 431 10.1007/s00253-005-0191-7 16283297

[B19] Sun Z Ramsay JA Guay M Ramsay BA Carbon-limited fed-batch production of medium-chain-length polyhydroxyalkanoates from nonanoic acid by *Pseudomonas putida* KT2440 Appl Microbiol Biotechnol 2007 3 69 77 10.1007/s00253-006-0655-4 17063330

[B20] Sun Z Ramsay JA Guay M Ramsay BA Enhanced yield of medium-chain-length polyhydroxyalkanoates from nonanoic acid by co-feeding glucose in carbon-limited, fed-batch culture J Biotechnol 2009 3 262 267 10.1016/j.jbiotec.2009.07.014 19632279

[B21] Van der Walle GAM De Koning GJM Weusthuis RA Eggink G Properties, modifications and applications of biopolyesters Adv Biochemical Eng Biotechnol 2001 3 263 291 10.1007/3-540-40021-4_9 11217415

[B22] Ward PG O’Connor KE Bacterial synthesis of polyhydroxyalkanoates containing aromatic and aliphatic monomers by *Pseudomonas putida* CA-3 Inter J Biol Macromol 2005 3 127 131 10.1016/j.ijbiomac.2005.01.001 15811466

[B23] Weatherburn MW Phenol-hypochlorite reaction for determination of ammonia Anal Chem 1967 3 971 974 10.1021/ac60252a045

